# *KRAS* mutations affect prognosis of non-small-cell lung cancer patients treated with first-line platinum containing chemotherapy

**DOI:** 10.18632/oncotarget.5607

**Published:** 2015-09-15

**Authors:** Mirko Marabese, Monica Ganzinelli, Marina C. Garassino, Frances A. Shepherd, Sheila Piva, Elisa Caiola, Marianna Macerelli, Anna Bettini, Calogero Lauricella, Irene Floriani, Gabriella Farina, Flavia Longo, Lucia Bonomi, M. Agnese Fabbri, Silvio Veronese, Silvia Marsoni, Massimo Broggini, Eliana Rulli

**Affiliations:** ^1^ Oncology Department, IRCCS - Istituto di Ricerche Farmacologiche Mario Negri, Milan, Italy; ^2^ Oncology Department, Fondazione IRCCS Istituto Nazionale dei Tumori, Milan, Italy; ^3^ Princess Margaret Cancer Center, University of Toronto, Toronto, Canada; ^4^ Oncology Department, Ospedale Fatebenefratelli e Oftalmico, Milan, Italy; ^5^ Oncology Department, Ospedale Papa Giovanni XXIII, Bergamo, Italy; ^6^ Molecular Pathology Unit, Niguarda Cancer Center, Ospedale Niguarda Ca' Granda, Milan, Italy; ^7^ Medical Oncology, Policlinico Umberto I, Rome, Italy; ^8^ Ospedale Belcolle, Viterbo, Italy; ^9^ Clinical Trials Coordination Unit, Institute for Cancer Research and Treatment, IRCCS, Candiolo, Italy

**Keywords:** KRAS, NSCLC, platinum, first-line

## Abstract

*KRAS* mutations seem to indicate a poor outcome in Non-Small-Cell Lung Cancer (NSCLC) but such evidence is still debated. The aim of this planned ancillary study within the TAILOR trial was to assess the prognostic value of *KRAS* mutations in advanced NSCLC patients treated with platinum-based first-line chemotherapy. Patients (*N* = 540), enrolled in the study in 52 Italian hospitals, were centrally genotyped twice in two independent laboratories for *EGFR* and *KRAS* mutational status.

Of these, 247 patients were eligible and included in the present study. The primary endpoint was overall survival (OS) according to *KRAS* mutational status in patients harboring EGFR wild-type.

Sixty (24.3%) out of 247 patients harbored *KRAS* mutations. Median OS was 14.3 months and 10.6 months in wild-type and mutated *KRAS* patients, respectively (unadjusted Hazard Ratio [HR]=1.41, 95%Confidence Interval [CI]: 1.03-1.94 *P* = 0.032; adjusted HR=1.39, 95%CI: 1.00-1.94 *P* = 0.050). This study, with all consecutive patients genotyped, indicates that the presence of *KRAS* mutations has a mild negative impact on OS in advanced NSCLC patient treated with a first-line platinum-containing regimen. Trial Registration: clinicaltrials.gov identifier NCT00637910

## INTRODUCTION

*KRAS* is a member of the *ras* gene family which encodes small G proteins with intrinsic GTPase activity. GTPase activity leads to protein inactivation and activates downstream effectors involved in multiple pathways including proliferation, differentiation and apoptosis. Point mutations occur in tumors resulting in the loss of intrinsic GTPase activity and consequently in the deregulation of cell proliferation signals [1].

*KRAS* is the most frequently mutated oncogene in Non-Small-Cell Lung Cancer (NSCLC) [2]. *KRAS* mutations are present in approximately 20% of lung adenocarcinomas, are more frequent in smokers, while infrequent in squamous cell tumors [3]. *KRAS* mutations in NSCLC are mainly missense in exon 2, codon 12 and 13, although other rare variants, such as codon 61, are also occasionally detected [4].

Although the *ras* gene was discovered almost thirty years ago, the role of *KRAS* mutations as prognostic and predictive markers in NSCLC cancer is still contentious [5, 6]. The available meta-analyses suggest that patients with wild-type KRAS have a better prognosis. On the other hand, the predictive role of KRAS mutations is uncertain caused by evidence mainly based on retrospective series with contradicting results likely due to patients selection bias, and therefore to the lack of proper planned randomized trials [7-11].

In addition, it seems that different types of *KRAS* mutations, according to the replaced bases, have a different role in carcinogenesis and drug response [12-15].

The aim of the study was to investigate, in terms of overall survival (OS) and progression free survival (PFS), the role of *KRAS* mutations in advanced *EGFR* wild-type NSCLC patients treated with first-line platinum-based chemotherapy.

## RESULTS

Between October 12, 2007 and March 13, 2012 we collected and genotyped for KRAS and EGFR 540 patients in the TAILOR trial [16]. Of these, 213 patients were not eligible for the present study for various reasons: adjuvant therapy (*N* = 177), missing data (*N* = 24), early stages at the time of first-line treatment (*N* = 6), KRAS status not evaluable (*N* = 3) and early death (*N* = 3). Eighty patients with tumor harboring EGFR gene mutations were also excluded. Of the remaining 247 eligible patients, 187 (76.8%) had wild-type *KRAS* tumor, whereas 60 (24.3%) had a tumor with a mutated *KRAS*. Nine different types of *KRAS* mutations were identified and the three most common were G12C (43.3%), G12V (23.3%) and G12D (10.0%) as reported in Table 1. G13 mutation isoforms (G13C and G13D) were seen in 6.7% (N = 4) of all mutated cases.

**Table 1:** Different type of *KRAS* mutations

*KRAS* mutations	N	%
G12A	6	10.0
G12C	26	43.3
G12D	6	10.0
G12F	2	3.3
G12R	1	1.7
G12S	1	1.7
G12V	14	23.3
G13C	2	3.3
G13D	2	3.3

**Figure 1:**
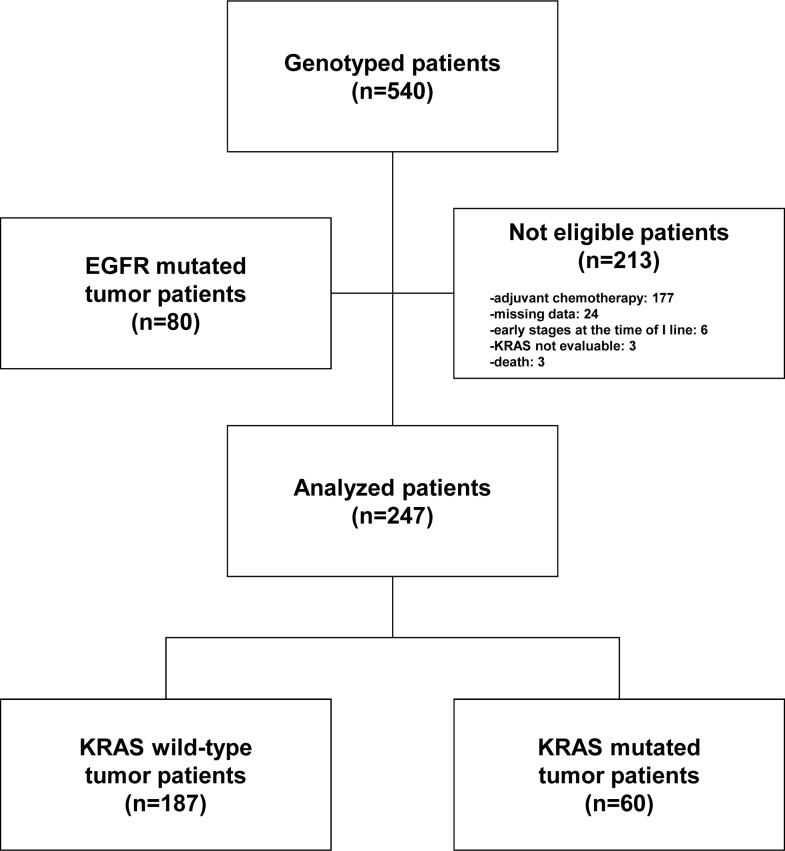
Patient CONSORT diagram

The CONSORT diagram is illustrated in Figure 1 whereas the baseline characteristics of the patients included in the study according to *KRAS* mutational status are illustrated in Table 2.

**Table 2:** Patient's characteristics

		*KRAS* wt		*KRAS* mutated		*P-value*
		*N*	%	*N*	%	
Patients		187	75.7	60	24.3	
Age of diagnosis	Median(25-75)	64.3 (57.4-70.3)		63.6 (56.0-67.7)		0.257
Sex	Male	134	71.7	46	76.7	0.449
	Female	53	28.3	14	23.3	
ECOG-PS	0	114	61.0	33	55.0	0.603^#^
	1	64	34.2	25	41.7	
	2	9	4.8	2	3.3	
Smoking	Never	42	22.5	4	6.7	0.006^#^
	Former smokers	88	47.1	30	50.0	
	Smokers	57	30.5	26	43.3	
Stage at diagnosis	IIIA	15	8.0	5	8.3	0.266^#^
	IIIB	24	12.8	4	6.7	
	IIIB wet	9	4.8	0	0.0	
	IV	139	74.3	51	85.0	
Grading	G1	7	6.7	2	6.5	0.329^#^
	G2	34	32.4	6	19.4	
	G3	63	60.0	23	74.2	
	Undifferentiated	1	1.0	0	0.0	
	unknown	82		29		
Histotype	Adenocarcinoma	123	65.8	51	85.0	0.038
	Squamous	49	26.2	5	8.3	
	Bronchoalveolar	3	1.6	0	0	
	Large cells	2	1.1	1	1.7	
	Mixed	6	3.2	3	5.0	
	Other	4	2.1	0	0	
Chemotherapy	Carboplatin	44	24.3	16	26.7	0.715
	Cisplatin	137	75.7	44	73.3	
	unknown	6		0		
Chemotherapy	Gemcitabine	105	57.1	22	37.9	0.019
	Vinorelbine	23	12.5	7	12.1	
	Pemetrexed	56	30.4	29	50.0	
	unknown	3		2		
Second-line	No random	82	43.9	30	50.0	0.406^1^
	Docetaxel*	55	52.4	17	56.7	0.679^2^
	Erlotinib*	50	47.6	13	43.3	
Third-line	None*	57	54.3	23	76.7	0.028^3^
	Pemetrexed**	18	37.5	3	42.9	0.905^2^
	Gemcitabine**	11	22.9	2	28.6	
	Vinorelbine**	13	27.1	1	14.3	
	Docetaxel**	4	8.3	1	14.3	
	Erlotinib**	2	4.2	0	0	

# Chi-square for trend

* percentage calculated on randomized patients

** percentage calculated on patients who received third-line treatment

1 comparison between randomized and not randomized patients

2 comparison among different treatments

3 comparison between treatment performed and no treatment

*KRAS* mutational status was associated with tumor histology (*P* = 0.038) and smoking habit (*P* = 0.006). The mutated *KRAS* subgroup of patients had, as expected, a higher percentage of adenocarcinoma histology (85.0% compared to 65.8% for mutated and wild-type respectively) and a lower prevalence of never smoker patients (6.7% compared to 22.5% for mutated and wild-type respectively). All the other characteristics were well balanced between the two groups.

All patients received platinum-doublet chemotherapy in the first-line setting with higher percentage of wild-type *KRAS* tumor patients receiving gemcitabine (57.1%) as compared to mutated tumor patients (37.9%). The latter received pemetrexed in a higher (50.0%) percentage compared to wild-type (30.4%). Vinorelbine option was less frequent but homogenously administered (12.5% and 12.1% in wild-type and mutated tumor patients respectively).

One-hundred and thirty-five patients were randomized in the main clinical trial. In particular, 52.4% and 56.7% of wild-type and mutated patients respectively were further treated with docetaxel in second-line treatment. On the other hand, 47.6% of wild-type and 43.3% of mutated patients received erlotinib. Among the randomized patients a higher percentage of patients with *KRAS* mutant tumor did not reach the third-line treatment (76.7%) compared to wild-type (54.3%) (*P* = 0.028).

### Survival outcomes

After a median follow-up of 52.5 months (95%Confidence Interval [CI] 42.0-64.7), 225 patients had progressed or died and 202 had died.

**Figure 2:**
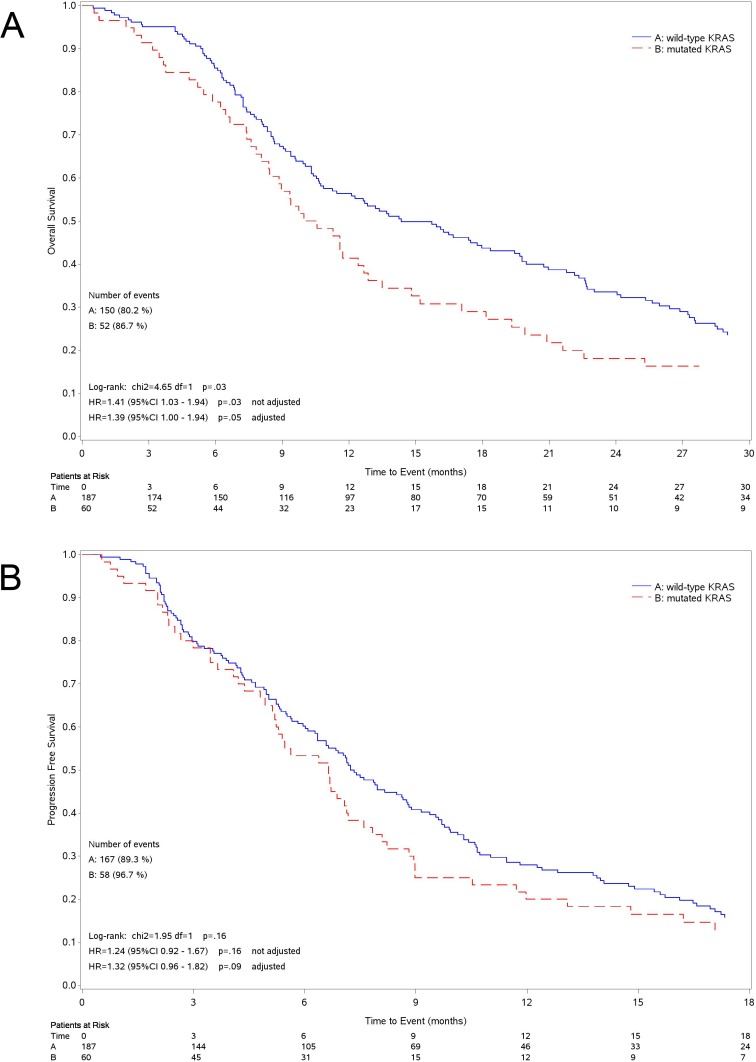
Kaplan-Meier curves for survival Curves for overall survival (A) and progression free survival (B) according to the KRAS status.

**Figure 3:**
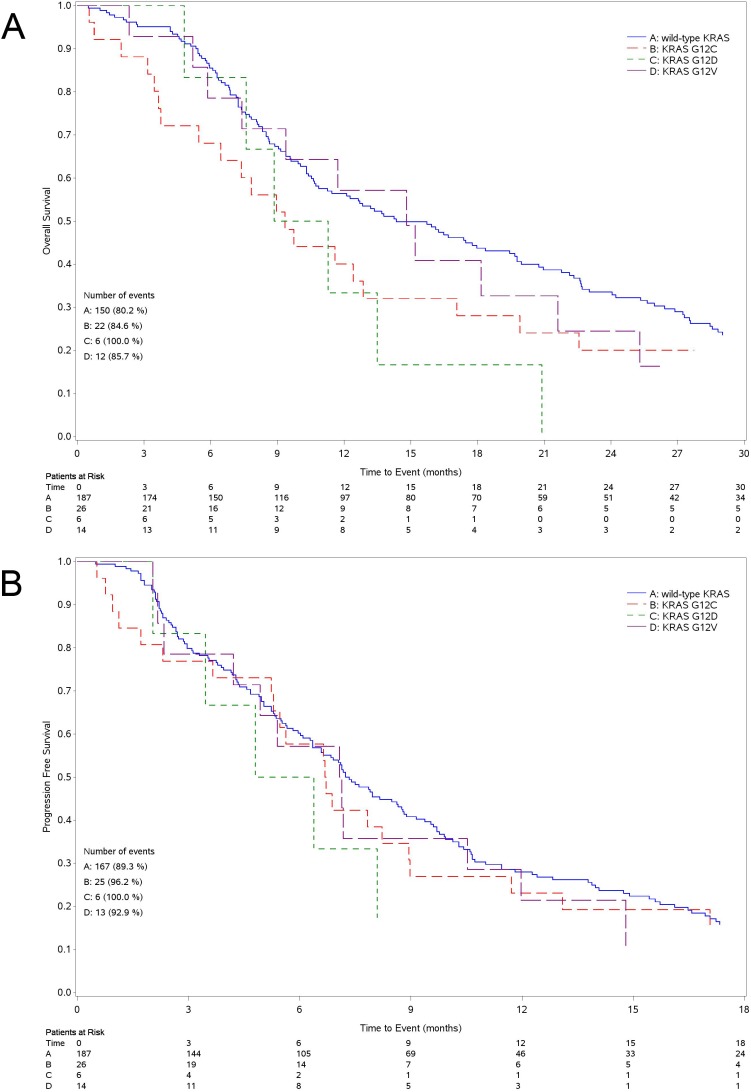
Kaplan Meier curves for survival Curves for overall survival **A.** and progression free survival **B.** according to the main different types of mutations (G12C, G12D, G12V).

Median OS was 14.4 months (95%CI 10.9-19.4) for patients with wild-type KRAS tumor and 10.6 months (95%CI 8.4-12.9) for those with mutant tumor (Figure 2A). The survival of patients with tumor harboring mutated *KRAS* was significantly lower than in wild-type group (unadjusted Hazard Ratio [HR] = 1.41 95%CI: 1.03-1.94 *P* = 0.032; adjusted HR = 1.39, 95%CI: 1.00-1.94 *P* = 0.050). The OS of patients expressing the three most common KRAS mutations, separately analyzed (G12C, G12D and G12V), was not different when compared to wild-type although all mutations had worsening trend (Figure 3A). The 4 patients harboring G13 *KRAS* mutations showed a median OS of 9.0 months.

ECOG-PS (HR = 1.79, 95%CI: 1.41-2.27 *P* < 0.001), sex (HR = 0.65, 95%CI: 0.47-0.89 *P* = 0.007) and tumor stage (HR = 1.18, 95%CI: 1.02-1.37 *P* = 0.024) were clinical factors significantly associated with OS. Risk estimate covariates are reported in Table 2.

Median PFS was 7.2 months (95%CI 6.3-8.8) for patients with wild-type KRAS tumor and 6.6 months (95%CI: 5.1-7.6) for those with mutant tumor (Figure 2B) (unadjusted HR = 1.24, 95%CI: 0.92-1.67 *P* = 0.164; adjusted HR = 1.32, 95%CI: 0.96-1.82 *P* = 0.092). As for OS, we could not find any statistical difference among patients with tumor harboring the three more common *KRAS* mutations, separately analyzed, and those with wild-type tumor (Figure 3B). Patients with G13 *KRAS* mutant tumor showed a median PFS of 3.9 months.

Tumor histology (HR = 0.70, 95%CI: 0.51-0.97 *P* = 0.033), ECOG-PS (HR = 1.53, 95%CI: 1.23-1.90 *P* < .001), and tumor stage (HR = 1.29, 95%CI: 1.11-1.48 *P* = 0.001) were statistically correlated with PFS. Risk estimate covariates are reported in Table 4.

**Table 3:** Prognostic evaluation of clinical and histopatological characteristics – Overall Survival

	HR	Lower 95% HR	Upper 95% HR	*P-value*
**Unadjusted**				
Age at diagnosis	0.99	0.98	1.01	0.385
ECOG-PS (2 vs 1 vs 0)	1.79	1.41	2.27	<.001
Histotype (squamous vs others)	0.78	0.55	1.10	0.149
Tumor stage (IV vs IIIB wet vs IIIB vs IIIA)	1.18	1.02	1.37	0.024
Tumor grade	1.28	0.94	1.73	0.113
Smoking (smoking vs former and not smoking)	1.31	0.92	1.88	0.135
Sex (F vs M)	0.65	0.47	0.89	0.007
Chemotherapy (cisplatin vs carboplatin)	1.13	0.82	1.56	0.448
Chemotherapy (gemcitabine vs vinorelbine)	1.45	0.90	2.33	0.133
Chemotherapy (pemetrexed vs vinorelbine)	1.70	1.03	2.80	0.038
*KRAS* (mut vs wt)	1.41	1.03	1.94	0.032
*KRAS* (12C vs wt)	1.42	0.91	2.22	0.128
*KRAS* (12D vs wt)	2.06	0.90	4.69	0.085
*KRAS* (12V vs wt)	1.24	0.69	2.24	0.471
**Adjusted**				
*KRAS* (mut vs wt)	1.39	1.00	1.94	0.050
ECOG-PS (2 vs 1 vs 0)	1.89	1.46	2.43	<.001
Tumor stage (IV vs IIIB wet vs IIIB vs IIIA)	1.18	1.01	1.37	0.042
Chemotherapy (gemcitabine vs vinorelbine)	1.32	0.80	2.17	0.271
Chemotherapy (pemetrexed vs vinorelbine)	1.61	0.95	2.74	0.077
Chemotherapy (cisplatin vs carboplatin)	1.17	0.84	1.63	0.345

**Table 4:** Prognostic evaluation of clinical and histopatological characteristics- Progression Free Survival

	HR	Lower 95% HR	Upper 95% HR	*P-value*
**Unadjusted**				
Age at diagnosis	0.99	0.97	1.00	0.080
ECOG-PS (2 vs 1 vs 0)	1.53	1.23	1.90	<.001
Histotype (squamous vs others)	0.70	0.50	0.97	0.033
Tumor stage (IV vs IIIB wet vs IIIB vs IIIA)	1.29	1.11	1.48	0.001
Tumor grade	1.09	0.81	1.46	0.578
Smoking (smoking vs former and not smoking)	1.13	0.81	1.58	0.471
Sex (F vs M)	0.90	0.67	1.20	0.463
Chemotherapy (cisplatin vs carboplatin)	1.15	0.84	1.56	0.383
Chemotherapy (gemcitabine vs vinorelbine)	1.76	1.12	2.78	0.015
Chemotherapy (pemetrexed vs vinorelbine)	1.78	1.10	2.86	0.018
*KRAS* (mut vs wt)	1.24	0.92	1.67	0.164
*KRAS* (12C vs wt)	1.07	0.70	1.63	0.754
*KRAS* (12D vs wt)	1.55	0.69	3.52	0.291
*KRAS* (12V vs wt)	1.22	0.69	2.15	0.493
**Adjusted**				
*KRAS* (mut vs wt)	1.32	0.96	1.82	0.092
ECOG-PS (2 vs 1 vs 0)	1.54	1.23	1.94	<.001
Tumor stage (IV vs IIIB wet vs IIIB vs IIIA)	1.26	1.08	1.46	0.004
Chemotherapy (gemcitabine vs vinorelbine)	1.67	1.04	2.68	0.034
Chemotherapy (pemetrexed vs vinorelbine)	1.64	1.00	2.71	0.051
Chemotherapy (cisplatin vs carboplatin)	1.26	0.92	1.74	0.151

## DISCUSSION

In the last 20 years many studies, including meta-analyses, with more than 8,000 patients considered have been published analyzing the prognostic and predictive role of *KRAS* mutations in NSCLC The majority of these results indicated *KRAS* as a negative prognostic and predictive marker [7-11, 17]. All these data were drawn from uncontrolled series and they included patients with different biological characteristics.

Our study confirms that a small negative prognostic effect of *KRAS* mutations can be observed in advanced NSCLC patients treated with a platinum-based doublet when EGFR mutant patients are excluded from the analysis. TAILOR results are superimposable to those found by Mascaux et al., the only meta-analysis performed on the role of *KRAS* in predicting efficacy of chemotherapy [10].

In addition, our epidemiological results are in line with literature: *KRAS* mutations are strongly correlated with smoking habit and adenocarcinoma histology and are mutually exclusive with EGFR mutations [3].

In a planned subgroup analysis of the main TAILOR study the *KRAS* mutations effect was not so clearly observed, although we cannot exclude a similar effect to the one presented in this paper with higher number of patients [18]. This difference may lay in the low statistical power of the TAILOR second-line subgroup analysis, or it may depend on a selected population with a better prognosis that can receive a second-line. Considering that a higher percentage of patients with a mutant *KRAS* tumor did not even underwent third-line treatment compared to wild-type we do not find these results in contrast with those previously reported by our group.

This analysis has some strengths and some limitations. The major strength is that this is the only pre-planned study in which *KRAS* status was evaluated in all consecutive patients included in the analysis and for whom *EGFR* mutations were also known. However, it is not possible to discriminate if the slightly worse prognosis of *KRAS* mutated patients is dependent on the poor prognostic effect of *KRAS* mutation or on a lesser response to the chemotherapy of these patients.

The treatment choice was done by physicians based on patients and disease characteristics therefore we cannot differentiate the final effect of cisplatin, carboplatin or other chemotherapeutic doublet agents according to *KRAS* status. On the other hand, the association between *KRAS* status and type of first-line chemotherapy can be explained by choice of treatment based on tumor histology, which is associated to the *KRAS* status. Nevertheless, the poorer outcome of mutated *KRAS* patients cannot be explained by a confounding effect related to second-line treatments given that wild-type and mutated *KRAS* patients were equally distributed in the two treatment arms in the main trial.

Results of the LACE-Bio pooled analysis, the largest pooled analysis encompassing 1543 patients from four large adjuvant studies (JBR.10, ANITA, CALGB, IALT), showed that there is no difference in terms of prognosis in early stage lung cancer patients with either wild-type or mutated *KRAS* [19]. Our different results could raise the hypothesis that *KRAS* mutations may play a different role in early and in advanced disease. This biological hypothesis could corroborate the evidence that, in advanced stages, *KRAS* is a condition necessary, but not sufficient to explain a more aggressive phenotype. Other additional factor(s) could contribute to this *KRAS* effect such as DNA repair capability. Advanced *KRAS* mutated tumors might have a DNA repair imbalance more pronounced than in early stages and our group is working actively on this issue [20, 21].

Due to small sample number, we are not able to elucidate any role for the three single most common *KRAS* mutations although the different variants seem to confer different OS when compared to the wild-type. Interestingly enough, preliminary preclinical data obtained in our laboratory in isogenic NSCLC cells differing only for the presence of *KRAS* mutations seem to suggest that the activity of cisplatin is different in cells expressing the different *KRAS* mutations [12].

Furthermore, the LACE-Bio pooled analysis pointed out that mutations in the codon 13 may confer a worse prognosis than others [19]. Although in TAILOR we have only an handful of G13 mutations, results seem to confirm this hypothesis.

The concept that not all *KRAS* mutations behave in the same way and that they differently impact on tumor progression has been addressed [12, 14, 15]. This data together with the indications reported in our study may suggest that proper trials need to be planned to define the role of the specific mutations in terms of response to treatment and tumor progression.

In conclusion, although *KRAS* showed a prognostic effect in first-line platinum-based treatment in advanced NSCLC, this study leads us to conclude that it is not warranted to test KRAS in clinical practice, at least until a specific targeted therapy is available for this group of patients. However, the potential mechanism of resistance to platinum-based therapies of these tumors should be further explored.

## MATERIALS AND METHODS

### Study design

TAILOR was a not-for-profit multicentre, open label, randomized trial, funded by the Italian Regulatory Agency AIFA and conducted in 52 Italian hospitals, comparing erlotinib versus docetaxel in second-line NSCLC and details have been published elsewhere [16]. Within the TAILOR trial we planned an ancillary study to assess the prognostic value of *KRAS* mutations in advanced NSCLC patients treated with a first-line platinum containing regimens.

Briefly, tumor samples from registered patients were histologically centrally reclassified according with the 2004 WHO classification. Suitable samples were genotyped in parallel by investigators in two independent laboratories using two different techniques: EGFR by Sanger's sequencing and restriction fragment length polymorphism whereas KRAS by Sanger sequencing and high-resolution melting analysis. Scorpion/ARMS technique was used for low-material samples. The Italian central authority and ethical review board at each participating Institution approved the protocol. The study complied with the declaration of Helsinki and was done in accordance with good clinical practice guidelines. Trial Registration: clinicaltrials.gov identifier NCT00637910.

### Patients and eligibility criteria

Participating centers registered all consecutive patients with NSCLC before or during first-line platinum-based chemotherapy as well as patients recurred after a first-line adjuvant platinum-based chemotherapy. Only those with both *EGFR* and *KRAS* status centrally determined were included in the trial. All patients received platinum-based chemotherapy in combination with either vinorelbine, gemcitabine or pemetrexed according to the physician's choice. Combinations with taxanes and with anti-EGFR agents were not allowed. Patients with EGFR mutations, early stages patients and patients receiving the adjuvant therapy were excluded from this analysis. All patients had an Eastern Cooperative Oncology Group (ECOG) Performance Status (PS) between 0 and 2 and were at least 18 years of age. Exclusion criteria included any evidence of serious co-morbidities that the investigator judged as a contraindication to the participation in the study, pregnancy and breast feeding. Patients were considered former smokers if they smoked more than 100 cigarettes in their life and they stopped this habit for at least one year at the time of diagnosis as used in most of smoking-habits analyses [22, 23]. All patients who were eligible for participation provided written informed consent with all applicable governing regulations before undergoing any study procedure.

### Statistical methods

The analysis was planned at the occurrence of 200 events, needed to detect a Hazard Ratio ≥1.60 (mutated *KRAS* vs. wild-type *KRAS*) assuming a *KRAS* mutation frequency of 25% with a statistical power of 80% and two-sided type I error of 5%.

The primary endpoint was OS defined as the time from the day of first-line treatment start to the date of death from any cause. The secondary endpoint was PFS defined as the time from the day of first-line treatment start up to the date of first progression or death from any cause, whichever came first. Patients who had not died or had disease progression at the date of study cutoff were censored at the last available information on status. Time-to-event data were described by the Kaplan-Meier curves. Cox proportional hazards models were used for univariate and multivariate analysis (adjusted for ECOG-PS, stage, type of first-line chemotherapy) to estimate and test demographic characteristics, clinical features, and biologic parameters for their associations with OS and PFS. We also evaluated OS and PFS according to the different subtypes of *KRAS* mutations.

Moreover we evaluated the association between the status of *KRAS* and clinical and histopathological characteristics by means of Chi-square test.

Results were expressed as HRs and their 95% confidence intervals and *P* values for two sided hypothesis test were reported.

All statistical analyses were carried out using SAS version 9.2 (SAS Institute, Cary, NC).
